# Revisiting Inherited Metabolic Diseases (IMDs) Through the Lens of Selective Autophagy

**DOI:** 10.1002/jimd.70259

**Published:** 2026-10-12

**Authors:** Marianna Maddaluno, Laura Cinque, Viviana Cetrangolo, Chiara De Leonibus

**Affiliations:** ^1^ Telethon Institute of Genetics and Medicine (TIGEM) Pozzuoli Italy; ^2^ Department of Clinical Medicine and Surgery Federico II University Naples Italy; ^3^ Department of Precision Medicine University of Campania “Luigi Vanvitelli” Naples Italy; ^4^ Department of Health Sciences University of Basilicata Potenza Italy

**Keywords:** bulk autophagy, inherited metabolic diseases (IMDs), selective autophagy, selective autophagy cargos (SACs), selective autophagy receptors (SARs)

## Abstract

Selective autophagy is increasingly recognized as a fundamental quality‐control mechanism that complements bulk autophagy by enabling the targeted degradation of specific intracellular cargos, including mitochondria, endoplasmic reticulum (ER), lipid droplets, glycogen, peroxisomes, ferritin, ribosomes, and protein aggregates. Cargo selectivity is mediated by selective autophagy receptors (SARs), which link damaged or superfluous substrates to the autophagic machinery through LC3/GABARAP interactions, thereby coordinating cargo sequestration. Recent studies have revealed substantial mechanistic differences between bulk and selective autophagy, highlighting the specialized signaling pathways, receptor networks, and transcriptional programs that regulate selective cargo clearance. Dysregulation of these pathways has emerged as a key contributor to the pathogenesis of inherited metabolic diseases (IMDs), a heterogeneous group of genetic disorders characterized by defective metabolism and multisystem involvement. This review summarizes the molecular mechanisms underlying major forms of selective autophagy, including mitophagy, pexophagy, glycophagy, lipophagy, ER‐phagy, and ferritinophagy. It also discusses their relevance across IMD classes, such as mitochondrial disorders, lysosomal storage disorders, and peroxisomal diseases, among many others. Furthermore, selective autophagy is highlighted as a potential therapeutic target, with modulation of autophagy receptors and signaling pathways offering promising strategies to restore cellular homeostasis and improve disease manifestations in IMDs.

## 1 Introduction

### 1.1 Bulk and Selective Autophagy—Mechanism Overview

Autophagy is a crucial cellular process that enables the breakdown and recycling of intracellular components by enclosing cytosolic substances in double‐membrane autophagic vesicles (AVs) that fuse with lysosomes for degradation [1]. Dysregulated autophagy is linked to several human diseases, driving interest in understanding its regulation for potential therapeutic development [2]. It is known that nutrient modulation through intermittent fasting and calorie restriction promotes lifespan by activating autophagy [3, 4], however the specific substrates responsible for these beneficial effects are still under investigation from many laboratories worldwide. Autophagy was once considered a bulk non‐selective process, but recent findings have uncovered selective forms of autophagy [5], in which specific substrates—referred to as selective autophagy cargos (SACs), including damaged mitochondria [6], endoplasmic reticulum (ER) fragments [7], lipid droplets [8], ribosomes [9, 10, 11, 12], peroxisomes [13], damaged lysosomes [14], and protein aggregates [15]—are targeted for degradation [5]. Although bulk and selective autophagy share the involvement of the core autophagy machinery, substantial mechanistic differences distinguish these two processes (Figure 1).

**FIGURE 1:**
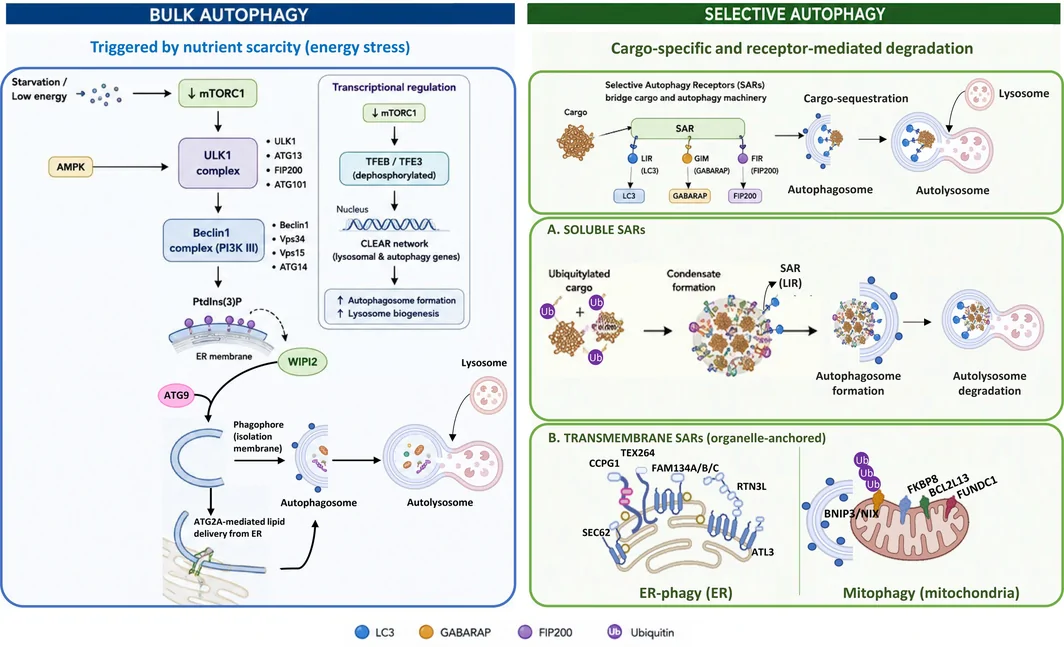
Bulk and selective autophagy: Distinct initiation mechanisms, shared core machinery. Left panel: Bulk autophagy is induced by nutrient deprivation and energy stress through mTORC1 inhibition and AMPK activation, leading to ULK1 complex activation, PI3K‐dependent phagophore nucleation, autophagosome formation, and lysosomal degradation. In parallel, TFEB/TFE3 activation promotes transcription of autophagy and lysosomal genes. Right panel: Selective autophagy is driven by the recognition of specific cargo by selective autophagy receptors, which link substrates to the autophagy machinery through interactions with LC3/GABARAP family proteins and FIP200. Soluble SARs (A) mediate the sequestration of both ubiquitylated and non‐ubiquitylated cargo, whereas transmembrane SARs (B) are constitutively associated with organelle membranes and promote organelle‐specific autophagic clearance. Despite their distinct initiation mechanisms, bulk and selective autophagy converge on the same core autophagy machinery to drive autophagosome formation and subsequent lysosomal cargo degradation.

**Bulk Autophagy**. During bulk autophagy, the biogenesis of autophagic vesicles is primarily triggered by reduced cellular energy levels, such as during starvation, and is regulated by nutrient‐sensing pathways like mTORC1 (mechanistic target of rapamycin complex 1) and AMPK (AMP‐activated protein kinase) [16]. During nutrient scarcity, inhibition of mTORC1 promotes the activation of the ULK1 complex, which consists of the scaffold proteins FIP200, ATG13, ATG101, and the serine/threonine kinase ULK1. Activation of this complex subsequently stimulates the class III PI3K nucleation complex (also known as the Beclin1 complex), composed of Beclin1, Vps34, Vps15, and ATG14. This complex generates a pool of phosphatidylinositol‐3‐phosphate (PtdIns(3)P) on the ER membrane, which locally recruits the WD repeat protein interacting with phosphoinositides (WIPI2), thus enabling autophagosome biogenesis [17]. Also, ATG9‐positive compartments function as initial pre‐autophagosomal structures that grow into flattened membrane cisternae, with the lipid transfer protein ATG2A driving lipid delivery from the endoplasmic reticulum to the expanding phagophore [18]. In addition, autophagosome biogenesis is also regulated at the transcriptional level. The inhibition of mTORC1 promotes the dephosphorylation of the MIT/TFE transcription factors (TFEB and TFE3), enabling their translocation to the nucleus, where they activate the CLEAR (Coordinated Lysosomal Expression and Regulation) gene network, thereby enhancing both autophagosome formation and lysosome biogenesis [19, 20, 21, 22, 23, 24] (Figure 1, left panel).

**Selective Autophagy**. Whereas bulk autophagy primarily functions as a metabolic adaptation mechanism during nutrient deprivation, selective autophagy operates as a quality‐control pathway that removes specific damaged or unnecessary cellular components. Unlike bulk autophagy, which is typically induced by nutrient starvation through inhibition of mTOR signaling, selective autophagy can occur even in the presence of active mTOR signaling [25]. In this context, cargo recognition and autophagosome biogenesis are tightly coupled through the action of selective autophagy receptors (SARs), which recruit the autophagy machinery to specific substrates and promote phagophore formation in their vicinity.

SARs act as molecular bridges between cargo and the expanding phagophore by simultaneously interacting with target substrates and components of the autophagy machinery, including members of the ATG8 protein family. These interactions are mediated by specific motifs, such as LC3‐interacting regions (LIRs), which bind ATG8 proteins of the LC3 subfamily, as well as GABARAP‐interacting motifs (GIMs) and FIP200‐interacting regions (FIRs), which mediate interactions with GABARAP proteins and FIP200, respectively. Together, these interactions promote the selective recruitment and sequestration of cargo by the autophagy machinery [26].

Following cargo recognition, the phagophore elongates around the targeted substrate through the recruitment of the core autophagy machinery shared with bulk autophagy (Beclin1 complex, Class III PI3K nucleation complex), ultimately enclosing the cargo within a double‐membrane autophagosome for lysosomal degradation [27]. Notably, cargo receptors can also function upstream of autophagosome biogenesis, promoting the sequestration and concentration of cargo proteins into higher‐order condensates, which subsequently become targeted for selective autophagic degradation [26].

Based on their localization and mechanism of action, SARs are broadly classified into two groups: (a) soluble SARs, which usually bind ubiquitylated cargos; and (b) transmembrane SARs that are constitutively associated with organelle membrane (Figure 1, right panel).

**Soluble SARs**. Ubiquitination is a post‐translational modification in which the small protein ubiquitin is covalently attached to target proteins, thereby influencing their fate and function. Depending on the type and context of ubiquitination, ubiquitinated proteins can be recognized and degraded by the Ubiquitin–Proteasome System (UPS), which primarily mediates the selective degradation of short‐lived or damaged proteins [28]. Ubiquitination also plays an important role in selective autophagy, where ubiquitinated cargo is recognized by autophagy receptors and delivered to autophagosomes for subsequent lysosomal degradation [28]. This process is mediated in part by soluble SARs, which act as molecular bridges between ubiquitylated cargo and the autophagy machinery.

Soluble SARs, including SQSTM1/p62, optineurin (OPTN), TAX1BP1, and NBR1, connect ubiquitylated cargo with the autophagy initiation machinery [29]. Among these receptors, SQSTM1/p62 and NBR1 play key roles in aggrephagy, where ubiquitylated protein aggregates are initially sequestered into condensates. TAX1BP1 is subsequently recruited to these condensates, promoting autophagosome membrane elongation around the cargo through its LIR domain [30]. This process is enhanced by Tank‐binding kinase 1 (TBK1)‐mediated phosphorylation of SQSTM1/p62, which facilitates TAX1BP1 recruitment and promotes efficient cargo engulfment and clearance through autophagy [30].

SQSTM1/p62 also functions in other forms of selective autophagy, including mitophagy, where TBK1‐dependent phosphorylation of SQSTM1/p62 promotes the sequestration of ubiquitylated mitochondria into autophagosomes [31]. Beyond damaged organelles, SQSTM1 condensates can capture diverse cellular components, including translation initiation factors [32], aggregation‐prone proteins [33], Vault complexes [34], and negative regulators of NRF2 signaling [35], highlighting their broad role in selective cargo recognition. SQSTM1/p62 also mediates ribophagy by recruiting ribosomes to the autophagy pathway during oncogene‐induced senescence [11]. More recently, TFEB activation was shown to promote the formation of SQSTM1/p62–NBR1–TAX1BP1‐positive ribosomal condensates, which recruit the ULK1 complex through FIP200, driving autophagosome formation and ribosome degradation [10].

Importantly, soluble SARs can also function through ubiquitin‐independent mechanisms. One example is the ferritinophagy receptor NCOA4, which binds ferritin to promote its autophagic degradation and iron release [36], a process relevant to iron‐overload disorders [37]. Similarly, the glycophagy receptor STBD1 mediates the autophagic degradation of glycogen [38], a pathway with significant relevance for cardiometabolic disorders [39].

**Transmembrane SARs**. Transmembrane SARs represent a distinct class of selective autophagy receptors anchored to organelle membranes, where they coordinate cargo recognition with autophagosome formation. These receptors include the ER‐phagy receptors, such as FAM134 family members (FAM134A/B/C), CCPG1, TEX264, ATL3, RTN3L, and SEC62, which mediate endoplasmic reticulum turnover [40], mitophagy receptors, including NIX/BNIP3, FUNDC1, AMBRA1, BCL2L13, FKBP8, PHB2, and NLRX1, which target mitochondria for degradation [41], and the lipophagy receptors, such as ATG14 [42], VPS4A [43], and ORP8 [44], for lipid droplet mobilization.

Recent studies have increasingly clarified how transmembrane SARs promote autophagosome biogenesis and facilitate cargo sequestration. Some receptors, such as FUNDC1 and BCL2L13, promote mitophagy through recruitment of the ULK1–FIP200 complex, whereas NIX/BNIP3 engages the WIPI–ATG13 machinery [18]. Interestingly, the ER‐phagy receptor TEX264 has been reported to utilize both mechanisms [45].

The activity of transmembrane SARs is regulated at both post‐translational and transcriptional levels. For instance, phosphorylation of the ER‐phagy receptors, FAM134C and TEX264, by the constitutively active kinase CK2 has been shown to regulate ER‐phagy activity [46, 47, 48]. In addition, ubiquitylation of FAM134B has been reported to promote ER‐phagy activation [49]. Beyond post‐translational regulation, transcriptional mechanisms also contribute to the control of SAR expression. A notable example is FAM134B isoform 2, a truncated variant of FAM134B whose expression is dynamically modulated across different physiological contexts. Initially identified as a starvation‐responsive isoform induced in the liver [50], FAM134B isoform 2 was subsequently found to be upregulated in chondrocytes upon FGF signaling [51]. More recently, its transcriptional regulation has been linked to skeletal muscle differentiation, with expression increasing during myogenesis [52].

Collectively, transmembrane SARs serve as central regulators of selective autophagy, coupling cargo recognition to autophagosome biogenesis through tightly regulated signaling networks. By orchestrating the selective turnover of organelles, they preserve organelle homeostasis, support cellular adaptation to physiological and environmental stresses, and maintain cellular integrity. Accordingly, dysregulation of SAR function has profound consequences for cellular homeostasis and is increasingly recognized as a contributing factor in a broad spectrum of human diseases. Among these receptors, members of the FAM134 family have emerged as key determinants of disease pathophysiology [53, 54, 55].

### 1.2 Autophagy With Its Variants in the Pathogenesis and Treatment of Inherited Metabolic Diseases

The “inherited metabolic diseases” (IMDs) represent a significant group of multi‐systemic disorders characterized by a progressive clinical course and caused by genetic defects in single enzymes or cofactors involved in metabolic pathways [56]. While the enzyme itself may be tissue‐specific, the loss of systemic metabolite circulation and tissue energy requirements results in damage of the primary site as well as of distant tissues, with the characteristic multi‐organ involvement of the disease. IMDs encompass a variety of distinct classes, ranging from Mitochondrial to Lysosomal Storage Diseases, among numerous others. A detailed hierarchical classification of the IMDs further discussed in the current Review is provided in Table 1.

**TABLE 1:** Correlation between selective autophagy pathways and inherited metabolic diseases, highlighting disease‐associated gene mutations and affected pathways in each condition.

IMDs—Classes	Gene	Protein	Function
Mitophagy			
*Mitochondrial diseases*			
Mitochondrial DNA depletion syndrome‐13	*FBXL4*	F‐box/LRR‐repeat protein 4	Phosphorylation‐dependent ubiquitination
PPTC7 Deficiency with hypomyelinating leukodystrophy	*PPTC7*	Protein Phosphatase Targeting CoQ7	Dephosphorylates the ubiquinone biosynthesis mitochondrial protein CoQ7
Coenzyme Q10 (CoQ10) deficiency	*COQ Genes*	CoQ biosynthetic proteins	Required for CoQ10 biosynthesis
Barth syndrome	*TAFAZZIN (TAZ)*	Phospholipid‐lysophospholipid transacylase	Cardiolipin remodeling; catalyzes transacylation reactions involved in the maturation and remodeling of mitochondrial cardiolipin
*Disorders of amino acid metabolism—Organic acidurias*			
Methylmalonic acidemia	*MMUT*	Methylmalonyl Coenzyme A mutase	Isomerization of methylmalonyl‐CoA (MMCoA) to succinyl‐CoA
Propionic acidemia	*PCCA*/*PCCB*	Propionyl‐CoA carboxylase (PCC)	Converts propionyl‐CoA to D‐methylmalonyl‐CoA
*Lysosomal storage disorders*			
Mucopolysaccharidoses			
Type I	*IDUA*	Alpha‐l‐iduronidase	Degradation of glycosaminoglycans (dermatan sulfate and/or heparan sulfate and/or chondroitin sulfate)
Type II	*IDS*	Iduronate 2‐Sulfatase	
Type IIIa (Sanfilippo syndrome)	*SGSH*	N‐Sulfoglucosamine Sulfohydrolase	
Type IIIc	*HGSNAT*	Heparan‐α‐glucosaminide N‐acetyltransferase	
MPS IVA	*GALNS*	N‐Acetylgalactosamine‐6‐sulfatase	
Type VI	*ARSB*	Arylsulfatase B	
Type VII	*GUSB*	Glucuronidase Beta	
Glycogen storage diseases (Pompe)	*GAA*	Acid α‐glucosidase	Glycogen degradation
Lysosomal lipid storage disorders			
Gaucher disease	*GBA*	Beta‐glucosidase	Glycosylceramide degradation
Fabry disease	*GLA*	Alpha‐galactosidase A	Degradation of glycosphingolipids
Niemann‐Pick disease	*NPC1*	NPC Intracellular Cholesterol Transporter 1	Cholesterol transport from the endo/lysosomal compartment
Pexophagy			
*Peroxisomal disorders*			
Zellweger spectrum disorders (ZSDs)	*PEX* genes	Peroxisomal biogenesis factors	Peroxisome biogenesis
Adrenoleukodystrophy	*ABCD1*	Peroxisomal membrane protein	Transport and beta‐oxidation of VLCFAs within peroxisomes
Glycophagy			
*Disorders of carbohydrate metabolism*			
Glycogen storage diseases			
Type Ia	*G6PC*	Glucose‐6‐phosphatase‐alpha	Hydrolysis glucose‐6‐p to glucose
Type Ib	*SLC37A4*	Glucose 6‐phosphate(p) translocase	Glucose‐6‐p Transport into ER lumen
Type II (Pompe disease)	*GAA*	Acid α‐glucosidase	Glycogen degradation
Lipophagy			
*Lysosomal storage disorders*			
Lysosomal lipid storage disorders		See above	
ER‐phagy			
*Lysosomal storage disorders*			
Niemann‐Pick disease		See above	
*ER‐storage disorders*			
Collagenopathies			
Kyphoscoliotic Ehlers‐Danlos Syndrome	*FKBP14*	FKBP Prolyl Isomerase 14	Folding of type III, VI and X collagens
Osteogenesis Imperfecta	*COL1A1*/*COL1A2*	Collagen Type I Alpha 1 Chain/Alpha 2 Chain	Fibril‐forming collagen found in most connective tissues (e.g., bone)
Congenital liver diseases			
Alpha‐1‐antitrypsin deficiency	*SERPINA1*	Alpha‐1‐antitrypsin	Irreversibly inhibits trypsin, chymotrypsin and plasminogen activator
Ferritinophagy			
*Disorders of iron metabolism*			
Hereditary hemochromatosis	*HFE*	Human homeostatic iron regulator protein	Control iron absorption by regulating the interaction of the transferrin receptor with transferrin
	*HJV*	Hemojuvelin	
	*TFR2*	Transferrin receptor protein 2	
β‐Propeller protein‐associated neurodegeneration (BPAN)	*WDR45*	WD Repeat‐Containing Protein 45 (WIPI4)	Interaction with ATG2A, involved in phagophore expansion and autophagosome maturation

### 1.3 The Implication of Selective Autophagy in the Pathogenesis of IMDs

Although the role of bulk autophagy in cellular homeostasis and disease has been extensively investigated, increasing evidence indicates that the selective clearance of specific intracellular cargo is equally critical for maintaining tissue function and metabolic integrity. IMDs are often characterized by the accumulation of toxic metabolites, damaged organelles, or aberrant macromolecular aggregates within particular cell types and tissues, highlighting the importance of cargo‐specific quality control mechanisms. Consequently, defects in selective autophagy pathways may not only exacerbate the primary metabolic insult but also contribute to disease progression.

This review provides an overview of the selective autophagy pathways shown to be impaired in IMDs, focusing on the nature of the accumulated cargo and its tissue‐specific distribution. The following sections examine the major forms of selective autophagy implicated in IMDs, including mitophagy, pexophagy, glycophagy, lipophagy, ER‐phagy, and ferritinophagy (summarized in Table 1 and Figure 2), discussing their underlying molecular mechanisms and physiological relevance. Particular emphasis is placed on how impaired selective autophagy promotes the accumulation of pathogenic substrates and dysfunctional organelles, thereby contributing to tissue dysfunction, disease progression, and clinical severity. Importantly, growing evidence suggests that molecular regulators of selective autophagy, while not necessarily the primary genetic cause of disease, can act as critical modifiers of pathological outcomes. Identifying these factors may reveal novel therapeutic strategies aimed at restoring cellular proteostasis and organelle quality control, ultimately slowing disease progression and improving clinical outcomes in patients with IMDs.

**FIGURE 2:**
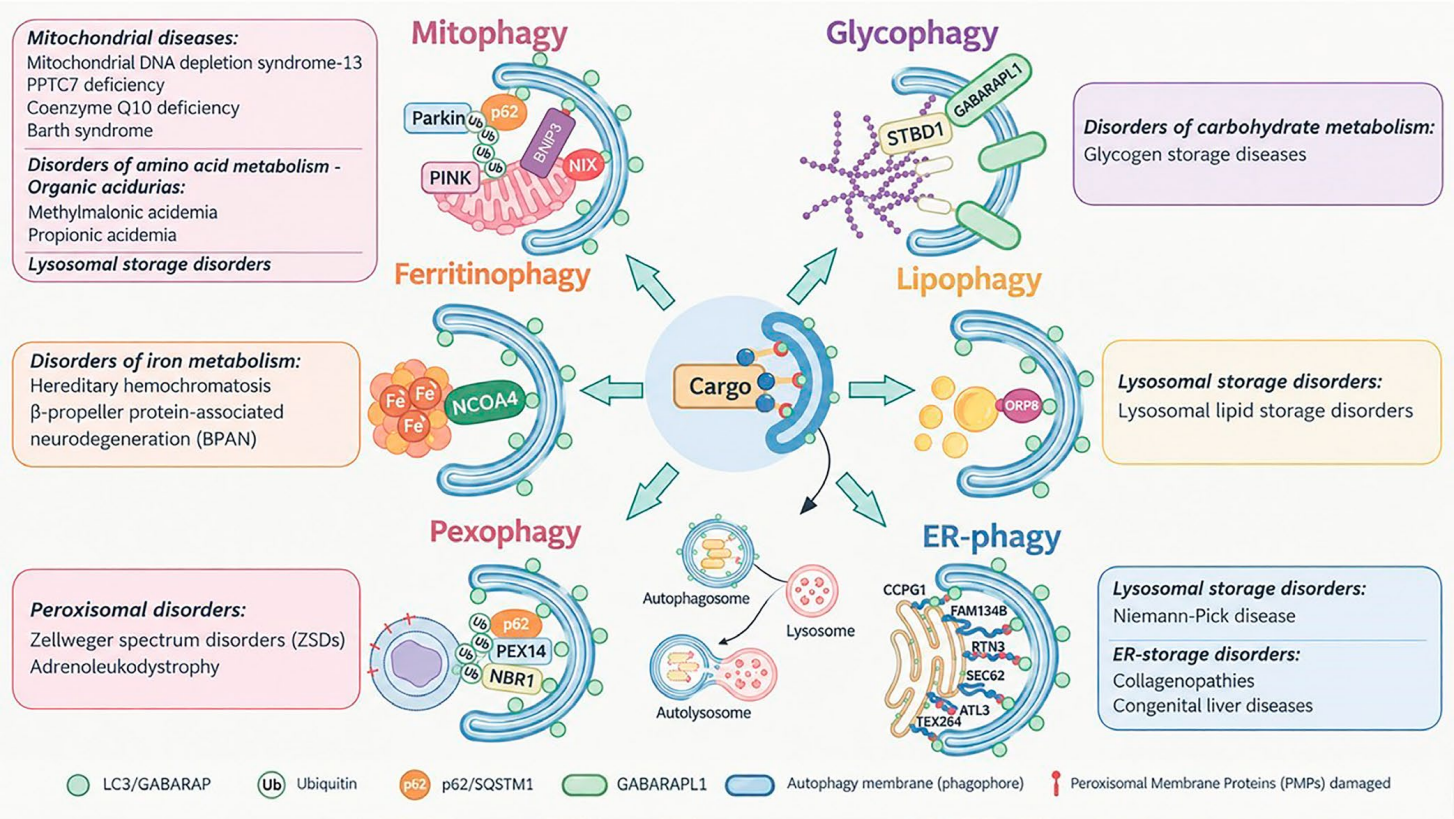
Selective autophagy pathways implicated in inherited metabolic disorders. Schematic overview illustrates the relationship between the dysregulation of selective autophagy pathways—including mitophagy, pexophagy, glycophagy, lipophagy, ER‐phagy, and ferritinophagy—and associated examples of IMDs. Ub, ubiquitin.

#### 1.3.1 Mitophagy

Mitophagy is an essential quality control mechanism that removes dysfunctional or excessive mitochondria by autophagy, maintaining cellular homeostasis and preventing the accumulation of damaged mitochondria [57]. This process is regulated by main complex and diverse regulatory mechanisms via either selective mitophagy receptors including BNIP3 and NIX or via PINK1/Parkin‐dependent mechanisms [58, 59]. Among these, the PINK1/Parkin pathway is the best‐characterized mechanism for maintaining mitochondrial integrity. Upon mitochondrial damage and loss of membrane potential, PINK1 (PTEN‐induced kinase 1) accumulates on the outer mitochondrial membrane, where it recruits and activates Parkin, an E3 ubiquitin ligase. Parkin activation critically depends on PINK1‐mediated phosphorylation. Specifically, PINK1 phosphorylates Parkin at Ser65 within its ubiquitin‐like domain, triggering a conformational change that converts Parkin from an inactive to an active state, thereby enabling its full functional activity [60, 61, 62]. Activated Parkin subsequently ubiquitinates multiple proteins on the outer mitochondrial membrane, labeling damaged mitochondria for recognition and degradation through the autophagy–lysosome pathway [63, 64].

Impairment of the mitophagy pathway has been identified in several groups of inborn errors of metabolism, including mitochondrial disorders, organic acidurias, and lysosomal storage diseases (LSDs) (overviewed in Table 1 and Figure 2 together with the other selective autophagy pathways discussed below).

Mitochondrial disorders represent a major category in which defective mitophagy has been increasingly implicated. Loss‐of‐function mutations in *FBXL4*, associated with Mitochondrial DNA depletion syndrome‐13, cause a severe mitochondrial disorder characterized by early‐onset lactic acidosis, encephalomyopathy, and developmental delay [65]. Mechanistically, *FBXL4* encodes a mitochondrial ubiquitin E3 ligase that negatively regulates mitophagy by promoting the degradation of the mitophagy receptors BNIP3 and NIX. Loss of FBXL4 results in excessive mitophagy, whereas deletion of either *BNIP3* or *NIX* in *Fbxl4*‐knockout mice attenuates mitophagy, restores metabolic homeostasis, and improves survival, highlighting these receptors as potential therapeutic targets in mitochondrial disease [66]. More recently, recessive loss‐of‐function mutations in the mitochondrial phosphatase *PPTC7* have been identified as the cause of a novel mitochondrial disorder characterized by hypomyelinating leukodystrophy, recurrent metabolic and lactic acidosis, anemia, and immune dysregulation. Similar to FBXL4, PPTC7 acts as a negative regulator of mitophagy by promoting the degradation of the mitophagy receptors BNIP3 and NIX. Accordingly, fibroblasts derived from affected individuals from two unrelated families exhibited excessive mitophagy, further supporting the pathogenic role of dysregulated mitophagy in mitochondrial disease [67]. Additional examples of mitochondrial disorders in which dysregulated mitophagy has been implicated include primary Coenzyme Q10 (CoQ10) deficiency and Barth syndrome. Multiple studies have reported alterations in mitophagy in these disorders [68, 69, 70, 71], further supporting a role for impaired mitochondrial quality control in their pathophysiology.

Second, a deregulation of the mitophagy pathway has also been described in Methylmalonic acidemia (MMA), the most common form of organic aciduria, due to deficiency of the mitochondrial methylmalonyl‐coenzyme A mutase (MMUT). In particular, MMUT deficiency limits PINK1/Parkin‐mediated mitophagy, leading to the accumulation of MMA‐damaged and/or dysfunctional mitochondria triggering cellular stress [72]. By performing drug‐disease network perturbation modeling, targetable biological pathways have been predicted, including redox homeostasis, whose modulation was found to preserve mitochondrial dysfunctions in patient‐derived cells and alleviate phenotype changes in MMUT‐deficient Zebrafish [72]. Similar findings have been reported in a propionic acidemia mouse model, in which PINK1/Parkin‐mediated mitophagy is also impaired, leading to the accumulation of dysfunctional mitochondria and further supporting defective mitophagy as a shared pathomechanism among organic acidurias [73].

Furthermore, impaired mitophagy together with mitochondrial dysfunction has been considered an important contributory factor to the pathophysiology of lysosomal storage diseases [74, 75, 76, 77], a group of approximately fifty rare inherited metabolic disorders [78]. Although each disorder derives from mutations in different genes encoding lysosomal hydrolases, LSDs share a common biochemical trait: the pathological accumulation of substrates within lysosomes resulting in lysosomal dysfunction. Depending on the specific enzyme deficiency, there is an accumulation of specific substrates, such as mucopolysaccharides in Mucopolysaccharidoses (MPS), glycogen in Glycogen storage disorders (Pompe Disease), sphingolipids in Lysosomal lipid storage disorders (Gaucher, Fabry, and Niemann‐Pick diseases), among many others [79]. Impaired mitophagy has been documented in a wide range of LSDs and affected tissues. These alterations have been observed: in neurons from Gaucher disease mice [80], skeletal muscle from Pompe disease models [76], adipose and brain tissues from MPS IIIA and IIIC models [81, 82], and liver [83], lungs [84], and chondrocyte tissues [85] from MPS II, MPS I, and MPS IVA models, respectively. Similar alterations have also been described in the brains of flies modeling MPS VII [86], and in fibroblasts derived from patients with MPS VI [87]. Mitophagy failure occurs well before the clinical symptoms become evident in LSD models, determining its prominent role in the onset of disease manifestations [88]. The loss of mitochondrial turnover that results from mitophagy decline leads to the accumulation of reactive oxygen species (ROS) and oxidative stress as well as a lack of ATP production and cellular energy [77, 80].

Overall, these findings highlight the central role of mitochondria in IMDs pathogenesis being a hub for oxidative phosphorylation and the production of cellular energy. Depending on the underlying defect, both the stimulation and normalization of mitophagy may represent beneficial strategies for restoring mitochondrial homeostasis. Promising therapeutic avenues include microRNA‐based approaches targeting OPA1 in mitochondrial diseases such as Stargardt disease type 1 (STGD1). OPA1 is a key regulator of mitochondrial dynamics, function, and mitochondrial quality control pathways, including mitophagy. Restoration of OPA1 expression through microRNA‐based therapy has been shown to improve mitochondrial integrity and ameliorate STGD1 phenotype [89]. As our understanding of the molecular mechanisms governing mitophagy continues to expand, the development of therapies aimed at fine‐tuning mitochondrial quality control may offer novel treatment opportunities across a broad spectrum of inherited metabolic diseases.

#### 1.3.2 Pexophagy

Peroxisomes are single‐membrane‐bound organelles that play essential roles in wide range of physiological processes, including glyoxylate detoxification and ether‐phospholipid biosynthesis. They contribute to maintaining oxidative homeostasis and lipid metabolism, particularly in the central nervous system, where they support neuronal and glial function, myelin synthesis, and membrane maintenance [90, 91, 92].

Peroxisome turnover is maintained through pexophagy, the selective autophagic degradation of excess or dysfunctional peroxisomes. Growing evidence indicates that this pathway plays an important neuroprotective role and contributes significantly to the pathology of neurological diseases [92, 93]. Pexophagy is mediated by SARs, primarily NBR1 and SQSTM1/p62, which contain both LIR and ubiquitin‐associated domains that facilitate the targeting of ubiquitinated peroxisomes to autophagosomes. In addition, nutrient deprivation induces a direct interaction between the peroxisomal membrane protein PEX14 and LC3, thereby facilitating pexophagy. The autophagy receptors NBR1 and SQSTM1/p62 further promote this process by enhancing the PEX14–LC3 interaction by inducing conformational changes that facilitate LC3 binding to PEX14 transmembrane domains [94]. More recently, Li et al. [95] identified an additional regulatory mechanism involving the interaction between PEX14 and the autophagy receptor OPTN, providing further evidence for a central role of PEX14 in coordinating the selective recognition and autophagic clearance of peroxisomes. These findings suggest that PEX14 may serve as an important molecular platform integrating distinct autophagy receptors to regulate pexophagy.

Dysregulation of pexophagy has been implicated in inherited peroxisomal diseases, particularly Zellweger spectrum disorders (ZSDs), a group of often severe peroxisome biogenesis disorders caused by mutations in *PEX* genes. ZSDs are characterized by defective peroxisome formation and function, leading to developmental abnormalities, hepatomegaly, visual impairment, and early infant mortality [96]. Several studies suggest that defective pexophagy contributes to disease progression in ZSDs, by promoting the accumulation of dysfunctional peroxisomes, increased oxidative stress, and altered lipid metabolism [97]. Notably, recent work by Germain et al. [98] revealed an important emerging mechanism in patient fibroblasts carrying the common pathogenic *PEX1* G843D variant, showing that excessive pexophagy promotes progressive peroxisome loss. This process sequesters components of the autophagic machinery at peroxisomes, limiting their availability for other selective autophagy pathways, including mitophagy and aggrephagy. The impairment of cellular quality control may contribute to the accumulation of damaged organelles and protein aggregates, further exacerbating disease pathology. Thus, in ZSDs, pexophagy dysregulation may not only result from impaired peroxisome homeostasis but may also actively compromise broader cellular proteostasis by competing for shared autophagic machinery.

Beyond Zellweger spectrum disorders, defective pexophagy is also implicated in the pathogenesis of Adrenoleukodystrophy (ALD). ALD is an inherited peroxisomal disorder, characterized by the accumulation of very long‐chain fatty acids (VLCFAs) in tissues, primarily affecting the adrenal cortex and white matter of the brain. This accumulation results from mutations in the *ABCD1* gene, which encodes a peroxisomal membrane protein essential for the transport and subsequent beta‐oxidation of VLCFAs within peroxisomes [99]. Dysregulation of pexophagy in ALD patients leads to an accumulation of dysfunctional peroxisomes, exacerbating the build‐up of VLCFAs and contributing to oxidative stress and neuroinflammation. Thus, impaired pexophagy disrupts cellular homeostasis and accelerates the neurodegenerative processes characteristic of ALD [97].

Therapeutic strategies aimed at enhancing pexophagy could potentially alleviate some of the metabolic disturbances in ZS and ALD. For instance, pharmacological agents that enhance pexophagy could help clear defective peroxisomes, thereby alleviating oxidative stress and restoring metabolic balance. Understanding the mechanisms regulating pexophagy and its dysregulation in IMDs opens new avenues for therapeutic intervention.

#### 1.3.3 Glycophagy

Glycophagy is the selective autophagic degradation of glycogen, a highly branched polysaccharide that functions as an energy reserve in the liver and skeletal muscle [39]. Starch‐binding domain‐containing protein 1 (STBD1) serves as the glycophagy receptor by binding glycogen through its carbohydrate‐binding module (CBM20) and targeting it for autophagic degradation [39, 100]. Besides the CBM20 domain, STBD1 contains seven binding domains for ATG8 partner proteins. STBD1 recruits glycogen to the forming phagosome (also known as glycophagosome), interacting with the GABA type A receptor associated protein like 1 (GABARAPL1), through the ATG8 interacting motif [38]. After autophagosome‐lysosome fusion, glycogen is degraded by the lysosomal acid α‐glucosidase (GAA). The role of STBD1 as a glycophagy receptor has not been extensively studied, but evidence suggests that the interaction between STBD1 and GABARAPL1 is crucial for regulating STBD1‐dependent glycophagy [38].

Glycophagy is disrupted in glycogen storage diseases (GSDs), a group of rare inherited metabolic disorders caused by pathogenic variants in genes involved in glycogen metabolism, including *G6PC*, *SLC37A4*, and *GAA*, among many others. A common feature of GSDs is the excessive intracellular accumulation of glycogen, primarily affecting the liver, skeletal muscle, or both [101, 102].

Among hepatic GSDs, GSD type Ia (GSDIa) and GSD type Ib (GSDIb) are autosomal recessive disorders caused by pathogenic variants in *G6PC* and *SLC37A4*, respectively. Both diseases are characterized by hepatomegaly and metabolic abnormalities, including hypoglycemia, lactic acidosis, and hyperlipidemia. GSDIa (Von Gierke disease) results from deficiency of glucose‐6‐phosphatase‐α (G6PC), impairing glycogen breakdown in the liver and leading to the accumulation of glycogen and triglycerides [103]. In addition, GSDIa is associated with defective autophagosome formation and impaired glycophagy, likely resulting from mTORC1 activation, AMPK inhibition, and reduced nuclear localization of TFEB [103, 104, 105]. Additionally, GSDIb is caused by variants in *SLC37A4* encoding for a glucose 6‐phosphate translocase (G6PT) which transports glucose‐6‐phosphate from the cytoplasm to the lumen of the ER during gluconeogenesis or glycogenolysis. Interestingly, it has been shown that SLC37A4 can negatively regulate mTORC1, which functions as an inhibitor of glycogen‐selective autophagy [101].

Glycophagy has also been implicated in Pompe disease (GSD type II), a lysosomal storage disorder caused by deficiency of acid α‐glucosidase. Loss of GAA activity results in excessive lysosomal glycogen accumulation, particularly in skeletal muscle, leading to progressive myopathy [76]. In the liver, the glycophagy receptor STBD1 mediates glycogen delivery to lysosomes through its CBM20 domain [106]. In contrast, studies in Pompe disease mouse models indicate that STBD1 plays a more limited role in skeletal muscle, where macroautophagy represents the predominant pathway for glycogen transport to lysosomes [107]. Nevertheless, preventing the delivery of cytosolic glycogen to lysosomes has been proposed as a potential therapeutic strategy for Pompe disease. In this context, STBD1 represents an attractive therapeutic target for limiting glycogen trafficking to lysosomes and reducing pathological glycogen accumulation [106].

#### 1.3.4 Lipophagy

Lipophagy is a selective autophagic pathway that targets lipid droplets (LDs), dynamic organelles that serve as the principal intracellular reservoirs of neutral lipids and play essential roles in lipid metabolism and energy homeostasis [108]. By mobilizing free fatty acids from stored lipids, lipophagy contributes to cellular energy production, particularly under conditions of nutrient deprivation [109].

Recent studies have identified several selective lipophagy receptors that couple LDs to the autophagic machinery. Among them, the oxysterol‐binding protein (OSBP)‐related protein 8 (ORP8) was recently characterized both in vitro and in vivo as a *bona fide* lipophagy receptor. ORP8 localizes to LDs and promotes their autophagic degradation through interactions with LC3/GABARAP family proteins in an AMPK‐dependent manner [44]. Additional lipophagy receptors have recently been identified, including ATG14, which mediates lipid droplet turnover through autophagy [42], and vacuolar protein sorting‐associated protein 4A (VPS4A), a selective lipophagy receptor characterized in both murine and human liver [43]. Starvation‐induced phosphorylation of VPS4A promotes lipophagy, thereby facilitating lipid mobilization and metabolic adaptation. VPS4A expression is markedly reduced in steatotic livers, suggesting a potential role in the pathogenesis of metabolic syndrome and related metabolic diseases [43].

While the role of lipophagy is well established in acquired metabolic conditions such as Metabolic dysfunction‐associated fatty liver disease (MAFLD) and hepatic steatosis, its contribution to inherited metabolic diseases remains less well defined. Nevertheless, impaired lipophagy has been proposed as a pathogenic mechanism in lysosomal lipid storage disorders, including Gaucher, Fabry, and Niemann–Pick diseases [110]. However, further research is needed, as neurons are particularly vulnerable to lipid accumulation, leading to early‐onset fatal neurodegeneration in affected patients, while treatment options remain limited. Specifically, future investigations should focus on understanding the involvement of lipophagy in lipid storage diseases and exploring the therapeutic potential of targeting this pathway to ameliorate neuronal symptoms.

#### 1.3.5 ER‐Phagy

The ER is a fundamental organelle involved in numerous cellular functions, including calcium storage and biosynthesis of lipids and proteins [111]. In this tightly regulated process, errors in protein synthesis due to genetic or environmental insults lead to protein misfolding and aggregation, resulting in ER stress and ultimately cell death. Mammals have developed evolutionary‐conserved ER quality control mechanisms to digest misfolded proteins and maintain cellular health [40, 112]. The best characterized is the ER‐associated degradation (ERAD) pathway, through which misfolded proteins translocate from the ER to the cytosol and are subsequently degraded via the proteasome. However, additional quality control mechanisms apply for misfolded ER clients that are not eligible for ERAD. These processes, collectively referred to as ER‐to‐lysosome–associated degradation (ERLAD), include ER‐phagy, microautophagy, and vesicular transport pathways [40, 112]. Among these mechanisms, ER‐phagy is a selective form of autophagy in which ER portions are selectively degraded through specialized ER‐phagy receptors that recognize specific ER regions and facilitate their sequestration into autophagosomes destined to lysosomal degradation. To date, a number of selective receptors has been identified including FAM134 family proteins (FAM134A/B/C), CCPG1, TEX264, SEC62, ATL3, and RTN3 [40]. Although they are structurally dissimilar and reside in different ER subregions (e.g., ER tubules or sheets), they all share one or more LIR domain that facilitates the binding of the cargo to LC3 proteins on the autophagosomal membrane, thus leading the delivery of ER fragments to AVs [7, 113, 114, 115].

ER‐phagy plays a prominent role in numerous crucial cellular functions including: (i) maintenance of ER turnover via the regulation of ER shape and size; and (ii) protein homeostasis with the selective degradation of ER luminal misfolded cargos. To date, the ER‐phagy receptors RTN3 and FAM134B have been implicated in the lysosomal degradation of a variety of misfolded proteins, including the lysosomal NPC intracellular cholesterol transporter 1 (NPC1), proinsulin, mutant proopiomelanocortin (POMC), pro‐arginine vasopressin (pro‐AVP), procollagen (PC), and mutant Z variant of α1‐antitrypsin (ATZ) [116, 117, 118, 119, 120]. Similarly, cargos identified for the ER‐phagy receptor CCPG1 include specific pancreatic ER luminal proteins [121].

Given its role in the clearance of a wide range of cellular cargos from the ER and lysosomal compartments, dysregulation of ER‐phagy has emerged as a contributing factor in multiple storage disorders, including lysosomal and ER‐storage disorders (ERSDs). In particular, defects in selective ER turnover impair the disposal of misfolded proteins, leading to their accumulation and disruption of cellular proteostasis. For example, in Niemann–Pick disease, an LSD caused by *NPC1* mutations, ER‐phagy contributes to the clearance of misfolded NPC1 variants retained in the ER. Impaired FAM134B‐dependent ER‐phagy has been observed in cellular and animal models as well as in patient‐derived samples, suggesting that defective ER turnover may exacerbate protein accumulation and disease progression [120].

ERSDs comprise a heterogenous group of inherited disorders characterized by accumulation of misfolded proteins in the ER [122, 123]. These disorders encompass a broad spectrum of conditions, including collagen‐related diseases, such as kyphoscoliotic Ehlers–Danlos syndrome (kEDS) and osteogenesis imperfecta (OI), congenital liver diseases caused by α1‐antitrypsin deficiency, early‐onset diabetes associated with mutant proinsulin or POMC, and neurohypophyseal diabetes insipidus caused by mutations in pro‐AVP, among others [123]. kEDS and OI are rare connective tissue disorders resulting from defects in collagen biosynthesis, folding, and maturation, leading to impaired extracellular matrix integrity [53, 124]. Similarly, α1‐antitrypsin deficiency represents the most common inherited cause of childhood liver disease. In this disorder, ATZ is inefficiently secreted and accumulates as insoluble polymers within the ER of hepatocytes, triggering chronic ER stress, inflammation, progressive liver damage, and an increased risk of hepatocellular carcinoma [125].

The accumulation of proteins at the ER not only overwhelms the cell's normal quality control mechanisms, but also leads to widespread cellular dysfunction, highlighting the importance of finding targeted therapies to mitigate the effects of ERSDs. One emerging approach is to selectively boost ER‐phagy. Notably, FAM134B‐dependent ER‐phagy is consistently upregulated across multiple cellular models of ERSDs, including kEDS, OI, and α1‐antitrypsin deficiency, suggesting that activation of this pathway represents a conserved adaptive response to the accumulation of misfolded proteins within the ER [53]. Importantly, overexpression of FAM134B in primary murine hepatocytes with α1‐antitrypsin deficiency significantly reduces the burden of insoluble ATZ polymer aggregates [53]. These findings provide proof‐of‐concept evidence that enhancing FAM134B‐mediated ER‐phagy may represent a promising therapeutic strategy for ERSDs by boosting the selective clearance of pathogenic protein aggregates and restoring ER proteostasis. Indeed, conducting a screening assay among FDA‐approved drugs, Tetrandrine and Fluphenazine have been identified as FAM134B transcriptional activators and potential ER‐phagy inducers. Fluphenazine, a phenothiazine compound similar to Carbamazepine, has been tested in a *Caenorhabditis elegans* model of α1‐antitrypsin deficiency [126]. Similarly, Tetrandrine, a calcium channel blocker known for its autophagy‐inducing properties [127] emerged as an ER‐phagy inducer capable of clearing misfolded cargo from the ER. The recent findings related to FAM134B‐mediated ER‐phagy offer prospects for drug repurposing in treating ERSDs diseases.

More recent evidence supporting the therapeutic relevance of autophagy‐mediated clearance of ER‐retained proteins comes from autosomal dominant tubulointerstitial kidney disease caused by mutations in the *UMOD* gene. In this disorder, mutant uromodulin accumulates within the ER of tubular epithelial cells, triggering ER stress and progressive kidney damage. Importantly, calorie restriction has been shown to enhance autophagy and promote the degradation of mutant uromodulin, thereby counteracting the primary pathogenic effects of *UMOD* mutations and significantly delaying disease onset and progression [128].

Collectively, these findings demonstrate that ER‐phagy plays a central role in maintaining ER proteostasis by facilitating the removal of aggregation‐prone proteins that escape conventional quality control mechanisms. The growing identification of disease‐associated ER‐phagy cargos and receptors, together with the emergence of pharmacological, genetic, and metabolic interventions capable of enhancing ER‐phagy, provides a strong rationale for targeting this pathway in ER‐storage disorders and other protein aggregation diseases.

#### 1.3.6 Ferritinophagy

Ferritinophagy is the selective autophagic degradation of ferritin and plays a key role in regulating cellular iron homeostasis. Central to this process is the autophagy receptor NCOA4 (Nuclear Receptor Coactivator 4), which binds to ferritin and facilitates its delivery to the autophagosome and subsequently to the lysosome for degradation with concurrent iron release. The ferritinophagy flux is tightly regulated by iron levels, which can modulate NCOA4 stability and therefore iron release. When cellular iron levels are elevated, iron‐bound NCOA4 interacts with the ubiquitin E3 ligase HERC2, marking NCOA4 for proteasomal degradation. This process reduces ferritinophagy. In contrast, when iron levels are low, the interaction between NCOA4 and HERC2 is inhibited, leading to NCOA4 stabilization. This results in increased ferritinophagy flux and iron release in the lysosome [36, 129].

Ferritinophagy has been implicated in inherited disorders of iron metabolism, including hereditary hemochromatosis (HH) and β‐propeller protein‐associated neurodegeneration (BPAN). In HH, mutations in iron‐regulatory genes (e.g., *HFE*, *HJV*, and *TFR2*) cause systemic iron overload and oxidative tissue damage [130, 131]. Recent studies showed that intestinal NCOA4‐mediated ferritinophagy contributes to iron accumulation, and its inhibition may protect against iron overload, suggesting a potential therapeutic target [132].

Ferritinophagy dysfunction is also central to a specific subtype of Neurodegeneration with Brain Iron Accumulation, namely BPAN (BPAN; OMIM #300894), caused by de novo mutations in the WD repeat domain 45 (*WDR45*) gene, encoding the autophagy protein WIPI4 [133]. BPAN is characterized by early‐onset global developmental delay followed by progressive neurological deterioration in adolescence or early adulthood, with brain MRI typically showing iron accumulation in the substantia nigra and globus pallidus [134].

At the molecular level, WIPI4 plays a central role in autophagy through its interaction with ATG2A, a lipid transfer protein required for phagophore expansion and autophagosome maturation [135]. Wdr45 knockout mice and Wdr45‐deficient cellular models have demonstrated consistent impairment of autophagy with disruption of autophagosome maturation and autophagosome–lysosome fusion, and defects in ferritinophagy with iron accumulation [134, 136]. In addition, BPAN patient‐derived fibroblasts exhibit defective ferritinophagy, as evidenced by impaired regulation of the ferritinophagy receptor NCOA4 in response to iron loading with ferric ammonium citrate (FAC). While control cells show a dose‐dependent increase in NCOA4 levels following FAC treatment, WDR45‐mutant fibroblasts fail to mount this adaptive response [137]. Neuronal models from patient‐derived iPSCs with *WDR45* mutation show shortened axon length, disruption of autophagy with significant iron accumulation contributing to neuronal apoptosis [138]. Collectively, these findings indicate that WDR45 dysfunction disrupts autophagy‐dependent pathways, including ferritinophagy, thereby impairing iron homeostasis, altering mitochondrial metabolism, and promoting ROS accumulation, ultimately contributing to neuronal degeneration in BPAN.

Although BPAN has long been considered a disorder primarily driven by autophagy impairment secondary to WIPI4 loss, emerging evidence supports the existence of additional autophagy‐independent mechanisms contributing to neurodegeneration. In particular, WIPI4 deficiency has been shown to trigger ferroptosis, an iron‐dependent form of regulated cell death characterized by excessive lipid peroxidation. Mechanistically, loss of WIPI4 disrupts the subcellular localization of ATG2, reducing its recruitment to phagophores while promoting its aberrant accumulation at mitochondria. This mislocalization induces mitochondrial membrane lipid peroxidation through the lipid‐transfer activity of ATG2, thereby initiating ferroptotic cell death. In turn, the resulting alterations in mitochondrial membrane lipid composition increase susceptibility to oxidative damage, further amplifying ferroptosis and contributing to progressive neuronal degeneration [139].

Understanding the in tricate mechanisms underlying ferritinophagy and its impact on iron metabolism is crucial for developing targeted therapies. For instance, pharmacological agents that selectively inhibit NCOA4 activity could ameliorate iron overload for the management of inborn errors of metabolism, thereby improving patient outcomes.

## 2 Conclusions and Future Perspectives

Autophagy with its variants plays a multifaceted role in the context of IMDs, influencing disease progression and providing a potential biological framework for disease mechanisms. Although substantial progress has been made in elucidating the role of selective autophagy in IMDs, important knowledge gaps remain.

Further studies are needed to define the causal contribution of selective autophagy defects to disease pathogenesis, unravel the molecular basis of tissue‐specific vulnerability, and clarify how distinct selective autophagy pathways interact with broader metabolic and quality‐control networks. A deeper understanding of the regulation of selective autophagy receptors and their signaling pathways will be essential for identifying novel disease modifiers and therapeutic targets. In addition, the development of reliable biomarkers and selective pharmacological modulators will facilitate the translation of these findings into clinical applications. Ultimately, continued investigation of selective autophagy is expected to provide new opportunities for restoring cellular homeostasis, slowing disease progression, and improving outcomes for patients with inherited metabolic diseases.

## Funding

The authors have nothing to report.

## Conflicts of Interest

The authors declare no conflicts of interest.

## Data Availability

The data that support the findings of this study are available on request from the corresponding author. The data are not publicly available due to privacy or ethical restrictions.
